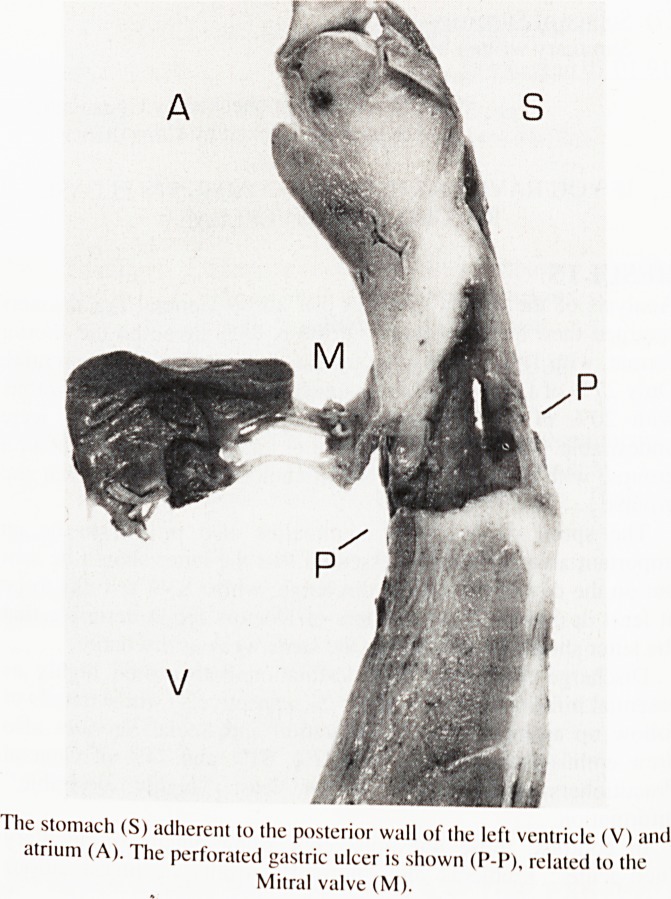# Perforation of a Peptic Ulcer in an Hiatus Hernia

**Published:** 1991-06

**Authors:** A. J. Hamer, E. Sheffield

**Affiliations:** Departments of Surgery and Pathology, Bristol Royal Infirmary, Marlborough Street, Bristol, BS2 8HW; Departments of Surgery and Pathology, Bristol Royal Infirmary, Marlborough Street, Bristol, BS2 8HW


### Perforation of a Peptic Ulcer in an Hiatus Hernia into the Left Ventricle

**A.J. Hamer, MB, ChB**
**E. Sheffield, BM MRC Path**
Departments of Surgery and Pathology,
Bristol Royal Infirmary,
Marlborough Street,
Bristol, BS2 8HW.

Gastric perforations are usually intraperitoneal, producing classic symptoms and signs. Perforation into adjacent organs, such as the colon, small bowel and aorta have been reported. Gastric ulcers in hiatus hernias have the potential to erode into mediastinal and thoracic structures such as the aorta, lung and bronchi. Penetration directly into the heart is very unusual.

We describe a case of a fatal perforation of an ulcer in an hiatus hernia, into the left ventricle of the heart.

### CASE REPORT

An 82 year old lady was admitted with a history of haematemesis and collapse. She had severe rheumatoid arthritis, which had been treated with piroxicam (Feldene, Pfizer Ltd.), and latterly with steroids. An ulcer, thought to have been duodenal had been seen on gastroscopy four months previously. She had been treated initially with ranitidine (Zantac, Glaxo Ltd.), and latterly with a six week course of omeprazole (Losec, Astra Pharmaceuticals). This had been stopped two months prior to admission.

On examination, she was pale and shocked with a tachycardia and a blood pressure of 100/60 mmHg. She was markedly tender in the epigastrium, without peritonism.

A diagnosis of gastrointestinal haemorrhage was made, and she was vigorously treated but deteriorated and died shortly after admission.

### PATHOLOGY

At post mortem the stomach contained a large amount of fresh blood and blood clot. There was a large hiatus hernia which was adherent to the posterior aspect of the heart. Opening the stomach revealed a four centimetre chronic gastric ulcer on the anterior wall. This had eroded through the full thickness of the stomach wall into the posterior aspect of the left ventricle. There was a three millimetre diameter perforation into the left ventricular cavity, the opening of the tract lying adjacent to the posterior cusp of the mitral valve (see Figure 1). Histology confirmed the presence of a benign ulcer.

### DISCUSSION

Large hiatus hernias are associated with gastric ulceration in about 30% of cases[1]. Ulcers within Hiatus Herniae penetrating the myocardium are exceedingly rare, and only seven such cases are reported in the literature[2,3,4].

Peptic ulcers of the intrabdominal stomach may also penetrate the myocardium, usually first becoming adherent to, and eroding through the diaphragm[5,6]. Twenty such cases were described by Porteous *et al.*[2], but in five of them previous gastric surgery had been performed.

In the present case, the ulceration was possibly related to the use of piroxicam and steroids. These anti-inflammatory agents have been strongly implicated in the pathogenesis of peptic ulcers with frequent fatal complications[7].

Erosion of an ulcer into the heart or a major blood vessel usually results in immediate fatal exsanguination. In some cases, however, an early warning bleed may result in early diagnosis and potential life saving surgery.

### REFERENCES

1. HILL, L.D. and TOBIAS, J.A. (1986) Paraesophageal Hernia. *Arch. Surg.* **96**, 735–744.
2. PORTEOUS, C., WILLIAMS, D., FOULIS, A. AND SUGDEN, B.A. (1984) Penetration of the left ventricular myocardium by benign peptic ulceration: Two cases and a review of the published work. *J. Clin. Pathol.* **37** (2), 1239–1244.
3. MELLET, J.S. and CILLIERS, P.H. (1987) Penetration of a gastric ulcer into the right ventricle. A complication of para-oesophageal hiatus hernia. *S. Afr. Med. J.* **72**, 44–45.
4. NICKELS, J. (1974) Peptic Ulcer in Hiatus Hernia penetrating the heart. *Br. J. Dis. Chest.* **68**, 273–278.
5. LAM, C.R., ANGULO, A.E. and PRIEST, R.J. (1975) Recurrent ulcer of the thoracic stomach penetrating the heart. Report of a case and review of the literature. *J. Thorac. Cardiovasc. Surg.* **69**, 835–838.
6. MACGILLIVRAY, J.B. AND MORGAN, M.N. (1971) Gastric ulcer penetrating the left ventricle. *Br. J. Surg.* **58**, 154–155.
7. ARMSTRONG, C.P., BLOWER, A.L. (1987) Non-steroidal anti-inflammatory drugs and life threatening complications of peptic ulceration. *Gut*, **28**, 527–532.

The stomach (S) adherent to the posterior wall of the left ventricle (V) and atrium (A). The perforated gastric ulcer is shown (P-P), related to the Mitral valve (M).

## Figures and Tables

**Figure f1:**